# Comparative study of a wearable intelligent sleep monitor and polysomnography monitor for the diagnosis of obstructive sleep apnea

**DOI:** 10.1007/s11325-022-02599-x

**Published:** 2022-03-26

**Authors:** Yanxia Xu, Qiong Ou, Yilu Cheng, Miaochan Lao, Guo Pei

**Affiliations:** 1 School of Medicine, South China University of Technology, Guangzhou, 510006 China; 2 Guangdong Provincial People’s Hospital, Guangdong Academy of Medical Sciences, Guangdong Provincial Geriatrics Institute, Guangzhou, 510080 China

**Keywords:** Obstructive sleep apnea, Wearable intelligent sleep monitor, Diagnosis, Polysomnography

## Abstract

**Purpose:**

Due to the lack of an objective population-based screening tool for obstructive sleep apnea (OSA), a large number of patients with potential OSA have not been identified in the general population. Our study compared an objective wearable sleep monitoring device with polysomnography (PSG) to provide a reference for OSA screening in a large population.

**Methods:**

Using a self-control method, patients admitted to our sleep center from July 2020 to March 2021 were selected for overnight PSG and wearable intelligent sleep monitor (WISM) at the same time. The sensitivity and specificity of the device for the diagnosis of OSA were evaluated.

**Results:**

A total of 196 participants (mean age: 45.1 ± 12.3 years [18–80 years]; 168 men [86%]) completed both PSG and WISM monitoring. Using an apnea–hypopnea index (AHI) ≥ 5 events/h as the diagnostic criterion, the sensitivity, specificity, kappa value, and area under the receiver operating characteristic curve of the WISM for OSA diagnosis were 93%, 77%, 0.6, and 0.95, respectively. Using an AHI ≥ 15 events/h as the diagnostic criterion for moderate-to-severe OSA, these values were 92%, 89%, 0.8, and 0.95, respectively. The mean difference in the AHI between PSG and the artificial intelligence oxygen desaturation index from the WISM was 6.8 events/h (95% confidence interval: − 13.1 to 26.7).

**Conclusion:**

Compared with the PSG, WISM exhibits good sensitivity and specificity for the diagnosis of OSA. This small, simple, and easy-to-use device is more suitable for OSA screening in a large population because of its single-step application procedure.

**Supplementary Information:**

The online version contains supplementary material available at 10.1007/s11325-022-02599-x.

## Introduction

Obstructive sleep apnea (OSA) is a common chronic sleep-related breathing disorder [1]. Patients with untreated OSA are at significantly increased risk of cardiovascular disease, stroke, cognitive dysfunction, and cancer [2–5], and their all-cause mortality is three times higher than that of the general population [6]. The number of patients with OSA worldwide has been reported to exceed 936 million, and this number has reached 176 million in China [7]. The Wisconsin Sleep Cohort Study investigated 4925 individuals aged 30–60 years. The study showed that individuals with mild (98% women and 90% men) and moderate and severe (93% women and 82% men) OSA were never diagnosed for this disease [8]. Similarly, the Sleep Heart Health Study showed that up to 91.7% of patients with suspected OSA with frequent snoring and sleepiness were undiagnosed [9]. These data indicate that OSA is a disease that is poorly understood by the public, and a large number of potential patients with OSA remain undiagnosed and untreated in a timely manner. Large clinical intervention trials have shown that continuous positive airway pressure (CPAP) therapy cannot prevent secondary cardiovascular events [10]. Hence, early diagnosis may be an effective way to prevent OSA-related complications and reduce morbidity and mortality.

The main diagnostic devices for OSA include polysomnography (PSG) and the type III portable monitor (PM). PSG is the “gold standard” for diagnosing OSA and grading its severity. A type III PM, which is based on the PSG, simplifies the monitoring channels and only contains three signals for respiratory airflow, chest and abdominal movement, and blood oxygen.  PM can be used to diagnose patients with moderate-to-severe OSA without comorbidities [11, 12]. These objective devices are all existing diagnostic methods for OSA, and all have high requirements for equipment as well as professional and technical personnel. These devices are also expensive and relatively inefficient, making them unsuitable for screening patients in a large sample of the population. Screening for OSA in the population mainly relies on various surveys/questionnaires [13]. However, the scales also require the participation of several professional and technical personnel and are time consuming. The results are also prone to subjective influence by the participants and researchers.

With the continuous development of monitoring technology, wearable sleep respiratory monitoring is more widely used [14]. One study used a finger pulse oximeter to diagnose patients with mild or greater and moderate to severe OSA; it showed the sensitivity and specificity of 80 and 70% and 86 and 91%, respectively. The diagnostic performance was not satisfactory, and we found that using the pulse oxygen clip for a long time caused finger discomfort during clinical practice [15]. Manoni et al. integrated photoplethysmography, accelerometer, microcontroller, and bluetooth transmission into a device that is required to be worn on the bridge of the nose, and hence, is prone to causing discomfort. This device was tested under laboratory conditions with a small sample size, and the feasibility and accuracy of screening for OSA in the population have not been verified [16]. Behar et al. and Al-Mardini et al. developed a smart phone-based device to screen for OSA. Although the device is a low-cost screening method, it requires wearing arm bands, microphones, and pulse oximeter [17, 18]. Wearing of the devices with external wires involves multiple steps that affect sleep. Further, older and less-educated individuals unfamiliar with electronics and medical devices face difficulties using them, and hence, such devices are not suitable for screening a large population. Therefore, an objective monitoring device that is small, lightweight, highly sensitive, less time-consuming, and can be used to screen hundreds of people every day remains to be developed. The wearable intelligent sleep monitor (WISM) is a portable device that can continuously monitor human oxygen saturation, heart rate, and body movement signals and analyzes oxygen desaturation index (ODI) using its own artificial intelligence (AI) algorithm. It is a compact device, has no external wires, and involves just one step to paste on the palm. The low difficulty of application can ensure the success rate of wearing and the integrity and validity of data collection. In this study, we evaluated the sensitivity and specificity of the WISM in diagnosing OSA.

## Methods

### Participants

The study was conducted in accordance with the tenets of the Declaration of Helsinki. Ethical approval for this study was obtained from the ethics committee of Guangdong Provincial People’s Hospital (No. GDREC2020221H(R1)).

The participants included patients with snoring as the main symptom who visited the sleep center at the Guangdong Provincial People’s Hospital, Guangzhou, China from July 2020 to March 2021. Participants of both sexes aged ≥ 18 years were included. The exclusion criteria were as follows: pregnancy; depression, anxiety, and other psychiatric illnesses and serious underlying diseases such as acute exacerbation of chronic obstructive pulmonary disease, acute myocardial infarction, unstable angina pectoris, congestive heart failure, and active infection.

### Collection of medical histories, symptoms, and signs

After obtaining informed consent and before conducting sleep monitoring, physicians at the sleep center privately collected basic demographic information (including sex, age, height, weight, and comorbidities) from participants in a quiet environment. The body mass index (BMI) (BMI = weight [kg] / height^2^ [m^2^]) was calculated for each participant. Participants also completed an assessment of sleep health based on OSA-related clinical symptoms.

### PSG and OSA diagnosis

Patients underwent an overnight PSG and WISM session at the sleep center simultaneously, conducted by experienced technicians, starting at 23:00 and ending at 6:00 the next day. The patients did not take a nap on the day of monitoring and did not drink tea, coffee, alcohol, or other beverages that would have interfered with their sleep. The scalp electrode was applied by an experienced technician in accordance with international standards [19]. The Alice 6 polysomnography (Philips Respironics Inc., USA) and Condi polysomnography (Grael PSG, Compumedics, Singen, Germany) apparatuses were used for OSA diagnosis. Nasal and oral airflow (nasal airflow pressure sensor, nasal and oral airflow thermal sensor), chest and abdominal movements, percutaneous oxygen saturation, snoring, body position, electroencephalogram recordings (F3, F4, C3, C4, O1, O2, M1, M2), mandibular electromyogram recordings, electrooculogram recordings, and data on other physiological indicators were obtained.

The results were interpreted manually by physicians trained in sleep medicine, in accordance with the American Academy of Sleep Medicine (AASM) standards on the Interpretation of Sleep and Related Events [11]. Apnea was defined as a ≥ 90% drop in nasal and oral airflow from the baseline and a continuous event of ≥ 10 s, with or without thoracic and abdominal respiratory movements. Hypopnea was defined as a decline in nasal and oral airflow of ≥ 30% from the baseline with a continuous event of ≥ 10 s, accompanied by a decrease in oxygen saturation of ≥ 3% or an event with arousal. The apnea–hypopnea index (AHI) was defined as the sum of the average number of apnea and hypopnea episodes per hour during sleep. The ODI obtained from the PSG was defined as the number of times oxygen saturation decreases by ≥ 3%/h during sleep. According to the PSG monitoring results, OSA was diagnosed using AHI of ≥ 5 and ≥ 15 events/h as thresholds. On the morning of the second day after monitoring, the patients were also required to complete a post-sleep monitoring questionnaire that collected information on the time to fall asleep, sleep at night, wake-up time, and abnormal conditions during the monitoring process.

### WISM use and analysis

The WISM (CloudCare Healthcare Ltd., Chengdu, China) is a portable sleep-monitoring device that monitors the blood oxygen saturation signal using a photoelectric reflex sensor (Supplementary Fig. S1). The original data were stored in a specific database. Based on the physical activity signals, level of pseudo-recognition, and automatic analysis of the AI software, the effective length and blood oxygen downtimes were monitored, and a report was subsequently generated. The monitoring sites were the palmar thenar major muscles. Veins, scars, spots, and locations with thick hair were avoided.

The main monitoring indices included the ODI, average oxygen saturation (AvSaO_2_) and lowest oxygen saturation (LSaO_2_), and percentage of sleep time spent with oxygen saturation below 90% (CT90%). The ODI obtained using the WISM was defined as a decrease in blood oxygen saturation of ≥ 3% or ≥ 4%. The AI algorithm automatically matched the degree of risk and selected ODI3% or ODI4%. The ODI referred to the total number of drops in oxygen saturation divided by the effective monitoring time.

### Statistical analysis

Data were analyzed using SPSS 23.0 software (IBM SPSS 23.0, Armonk, NY, USA). Continuous variables are presented as means ± standard deviations (for normal distributions) or medians and interquartile ranges (for non-normal distributions). The sensitivity, specificity, positive predictive value, and negative predictive value of the WISM were calculated using the AHI and ODI thresholds of ≥ 5 and ≥ 15 events/h obtained from the PSG, and the consistency of the two methods was tested using the kappa value. The correlation between the ODI, obtained from the WISM, and the AHI and ODI, obtained from the PSG, was analyzed by the Pearson correlation. The consistency of the WISM and PSG results was determined using the Bland–Altman method. The best cutoff WISM values for OSA diagnosis were determined by examining the area under the receiver operating characteristic (ROC) curve.

## Results

### Participants’ characteristics

A total of 196 participants completed the PSG and used the WISM. Of them, 168 patients were men (86%), 28 were women (14%), and the average age was 45.1 ± 12.3 years (18–80 years). The average BMI was 26.3 ± 3.7 kg/m^2^. A total of 26% of the patients had obesity (BMI of ≥ 28 kg/m^2^). According to the PSG results, 39 patients (20%) had mild OSA, and 135 (69%) had moderate-to-severe OSA (Table 1).

**Table 1:** Characteristics of the study participants

Characteristics	*n* = 196
Age in years	45.1 ± 12.3
Sex (*n*, %)	
Men	168 (86)
Women	28 (14)
Height (m)	1.7 ± 0.1
Weight (kg)	76.4 ± 14.0
BMI (kg/m^2^)	26.3 ± 3.7
Sleep stages, %	
N1	13.0 ± 11.2
N2	57.3 ± 14.3
N3	14.4 ± 11.2
REM	15.3 ± 7.8
Severity of OSA (%)	
Non-OSA	22 (11)
Mild OSA	39 (0)
Moderate-to-severe OSA	135 (69)

*BMI* body mass index, *OSA* obstructive sleep apnea, *REM* rapid eye movement

### Sensitivity and specificity of WISM in diagnosing OSA

Table 2 shows the sensitivity, specificity, positive predictive value, negative predictive value, positive likelihood ratio, negative likelihood ratio, and accuracy of the WISM in diagnosing OSA at the AHI ≥ 5, ≥ 15 and ODI ≥ 5, ≥ 15 thresholds when compared with the PSG. The sensitivity was high when the AHI was ≥ 5 events/h (sensitivity = 93%, specificity = 77%), and both sensitivity and specificity were high when the AHI was ≥ 15 events/h (sensitivity = 92%, specificity = 89%). The sensitivity, specificity, and accuracy values for the WISM among patients with obesity who had OSA (BMI of ≥ 28 kg/m^2^) were 98%, 100%, and 98%, respectively, as shown in Table 2.

**Table 2:** Performance metrics of the wearable intelligent sleep monitor at apnea–hypopnea index and oxygen desaturation index thresholds of ≥ 5 and ≥ 15 events/h

Screening threshold	Participants above the threshold	Sensitivity (%)	Specificity (%)	PPV (%)	NPV (%)	p-LHR	n-LHR	Accuracy (%)	*κ*	AUC
AHI ≥ 5 events/h	174	93	77	97	59	4.1	0.1	91	0.6	0.95
AHI ≥ 15 events/h	135	92	89	95	83	8.0	0.1	91	0.8	0.95
ODI ≥ 5 events/h	169	93	67	95	62	2.8	0.1	90	0.6	0.94
ODI ≥ 15 events/h	132	94	89	95	88	8.6	0.1	92	0.8	0.97
Patients with obesity										
AHI ≥ 5 events/h	56	98	100	100	67		0.0	98	0.8	1
AHI ≥ 15 events/h	51	92	100	100	64		0.1	93	0.7	0.95
ODI ≥ 5 events/h	54	98	50	96	67	2.0	0.0	95	0.5	0.99
ODI ≥ 15 events/h	50	94	100	100	73		0.1	95	0.8	1

*PPV* positive predictive value, *NPV* negative predictive value, *p-LHR* positive likelihood ratio, *n-LHR* negative likelihood ratio, *AHI* apnea–hypopnea index, *ODI* oxygen desaturation index

### ROC curve for OSA diagnosis using the WISM cutoff values

**Fig. 1:**
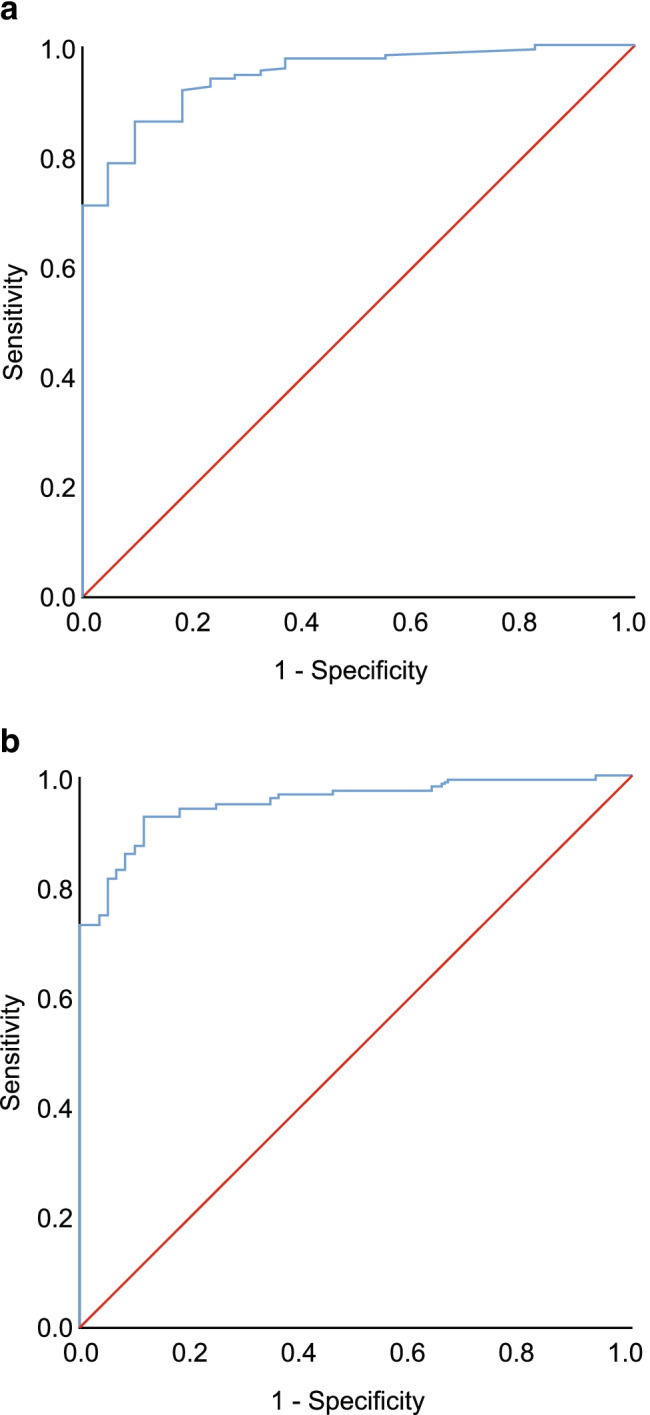
ROC curves for OSA diagnosis using the WISM. **a** ROC curve for OSA diagnosis using the WISM at an AHI threshold of ≥ 5 events/h. **b** ROC curve for OSA diagnosis using the WISM at an AHI threshold of ≥ 15 events/h. *OSA* obstructive sleep apnea, *ROC* receiver-operating characteristic, *WISM* wearable intelligent sleep monitor, *AHI* apnea–hypopnea index

When an AHI of ≥ 5 events/h was used as the criterion for diagnosing OSA, the area under the ROC curve was 0.95 (*P* < 0.001, 95% confidence interval [CI] = 0.910–0.983), the best cutoff value was 7, the sensitivity was 86%, and the specificity was 91%. When an AHI of ≥ 15 events/h was used as the criterion for diagnosing OSA, the area under the ROC curve was 0.95 (*P* < 0.001, 95% CI = 0.922–0.979), the best cutoff value was 12, the sensitivity was 93%, and the specificity was 88% (Fig. 1).

### AHI and WISM ODI conformance analysis

**Fig. 2:**
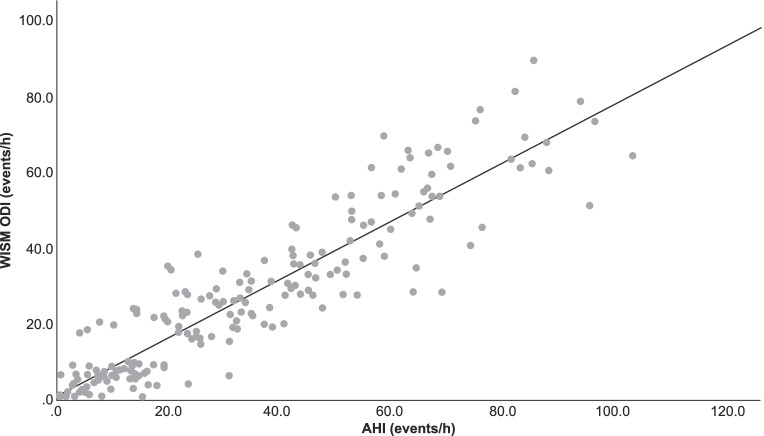
Linear correlation analysis of ODI and AHI (*R*^2^ = 0.843, *P* < 0.001). *ODI* oxygen desaturation index, *AHI* apnea–hypopnea index, *PSG* polysomnography

**Fig. 3:**
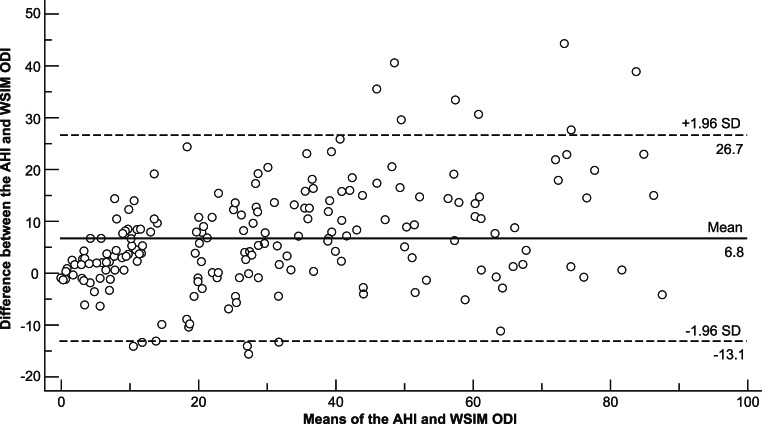
Bland–Altman consistency test results for the ODI calculated from the WISM and the AHI calculated from the PSG. The mean difference between the ODI from the WISM and AHI from the PSG was 6.8 (consistency limit: − 13.1–26.7 [*N* = 196)]. *ODI* oxygen desaturation index, *AHI* apnea–hypopnea index, *PSG* polysomnography, *WISM* wearable intelligent sleep monitor

The kappa coefficients of the ODI from the WISM at different thresholds are shown in Table 2. The ODI had a strong correlation with the AHI and ODI from the PSG (*R*^2^ = 0.843, *R*^2^ = 0.845) (Fig. 2; Supplementary Fig. S2). The Bland–Altman curves for the ODI from the WISM and AHI from the PSG are shown in Fig. 3; 93% (182/196) of the scatter points lie within the 95% limits of agreement (LOA). The Bland–Altman curves for the ODIs from the WISM and PSG are shown in Supplementary Figure S3, where 96% (188/196) of the scatter points lie the 95% LOA.

## Discussion

The results of this study showed that there was a strong correlation and consistency between the ODI obtained from the WISM and the AHI and ODI obtained from the standard PSG, suggesting that as a screening device, the WISM showed good diagnostic performance. Our results support the use of a highly accurate and convenient monitoring tool to screen for OSA in a large population, allowing a large number of potential patients with OSA to be diagnosed and treated in a timely manner.

In our study, with the AHI ≥ 5 and PSG ODI ≥ 5 as the diagnostic threshold, the sensitivity was high, but the specificity was slightly lower, which may be related to the small sample size of the patients who did not have OSA (22/196, 27/196). In contrast, with the AHI ≥ 15 and PSG ODI ≥ 15 as the diagnostic threshold, high sensitivity and good specificity were observed. For all of these threshold, the areas under the ROC curve (AUC) were ≥ 0.9; compared with the sensitivity, specificity, and AUC of the single-channel monitors in previous studies [20], the device has strong diagnostic value to screen for OSA with corresponding thresholds. In particular, with the AHI ≥ 15 threshold showing a high positive likelihood ratio and a low negative likelihood ratio, the best cutoff value for diagnosing moderate-to-severe OSA was 12, with a sensitivity and specificity of 93% and 88%, respectively. This result is consistent with the ODI defined for the moderate-to-severe OSA in a large-scale interventional study by McEvoy et al. [10]. When comparing the ODI obtained from the WISM to that of the PSG, the sensitivity of WISM, at the PSG ODI ≥ 5 and ≥ 15, were 93% and 94%. These were higher than the sensitivity of AHI at the corresponding thresholds. When the ODI was ≥ 15, the AUC reached 0.97, indicating that some patients’ ODI obtained from the WISM underestimated the AHI. This may be related to the definition of apnea and hypopnea; apnea may not be accompanied by a decrease in oxygen saturation, while hypopnea is a condition that shows decreased oxygen saturation or microarousal [11]. Therefore, patients with a negative diagnosis of WISM but OSA symptoms should be tested using the PSG. The specificity of WISM when PSG ODI ≥ 5 was lower as compared to when PSG AHI ≥ 5. This may be due to the small sample size of patients who did not have OSA, and can be further studied in a large population.

The WISM ODI < 7 was used as the threshold to screen for patients without OSA, and only two cases (0.1%) were misclassified. Among the 45 WISM-diagnosed patients with mild OSA, one remained undiagnosed, while four had moderate and non-severe OSA according to the AASM OSA diagnostic criteria [11]; among the 132 WISM-diagnosed patients with moderate and severe OSA, one remained undiagnosed, while six showed mild OSA. This indicates low false-positive and misdiagnosis rates of the WISM, highlighting its capability to exclude patients without OSA, which can help to avoid economic loss, psychological stress, and unnecessary use of medical and healthcare resources caused. The WISM calculates the ODI using effective monitoring time instead of sleep time as the denominator, and hence, may underestimate the condition of OSA. PSG and follow-up should be performed for patients with OSA and insomnia. Patients with obesity are an important subgroup of patients with OSA, and the pathophysiology of OSA in patients with obesity differs from that in the general population [21]. We also observed that the sensitivity and accuracy of the WISM among the obese patients were high, with good consistency.

A recent study showed that the sensitivity/specificity (AUC) of using wearable wrist worn monitor to estimate apnea in patients with mild, moderate, and severe OSA were 77/72% (0.84), 62/91% (0.86), and 46/98% (0.85), respectively. The diagnostic performance of the devices was moderate, with slightly lower kappa values than that of the WISM [22]. Nigro et al. used blood oxygen saturation and airflow of the dual-channel portable monitors to evaluate suspected OSA and found that manual analysis was superior to the autoscoring set up by the device (AUC 0.923 vs. 0.87) [23]. However, this device has airflow lead, which is not simple enough and requires manual scoring to achieve the better diagnostic ability; it is not suitable for screening a large sample population. Electrocardiogram signal is closely related to respiration, and has been used to diagnose OSA [24]. The work conducted by Arikawa et al. have used more convenient mobile wearable heart rate sensors and R-R intervals (RRI) to analyze and predict OSA risk [25]. However, the diagnostic test is on a small sample of people, and the performance is not clear. The WISM directly monitors blood oxygen saturation, and the AI algorithm analyzes the ODI. It is small and sometimes easy to lose, but with good accuracy. It has no external lead and no sense of wearing, which can improve the participation rate of people with a high risk of OSA and make it possible to screen for OSA in a community or a large sample population.

This study had limitations. All of the participants were patients attending a sleep center, and did not represent the general population. Furthermore, the device does not distinguish between obstructive apnea and central apnea due to the absence of airflow and thermal leads. The device is also unable to monitor sleep time. Therefore, further research and development of such devices are needed in the future.

In conclusion, at present, many patients with potential OSA remain unidentified, and OSA screening in the general population mainly relies on responses to questionnaires. Thus, there is a need for an objective device that is small, lightweight, less time-consuming, and capable of screening hundreds of people a day. Compared with PSG, WISM exhibits good sensitivity and specificity, and the operation of the device requires only a single step. Our findings suggest that this device may aid in objective, rapid screening of large numbers of patients. 

## Supplementary Information

Below is the link to the electronic supplementary material.

Supplementary file1 (PDF 302 KB)

## Data Availability

The detailed participant data are available from the corresponding author upon reasonable request.
