# Supplementary material for: Comparative study of a wearable intelligent sleep monitor and polysomnography monitor for the diagnosis of obstructive sleep apnea
Source: Sleep Breath. 2022 Mar 26;27(1):205–12. doi: 10.1007/s11325-022-02599-x (PMC9992231; doi:10.1007/s11325-022-02599-x)
Supplement: Supplementary file 1 — Supplementary file1 (PDF 302 KB) [file 11325_2022_2599_MOESM1_ESM.pdf]

Supplementary Information

Title: Comparative study of a wearable intelligent sleep monitor and polysomnography monitor for the diagnosis of obstructive sleep apnea

[Author names]
Yanxia Xu,[1] Qiong Ou,[2] Yilu Cheng,[1] Miaochan Lao,[2] Guo Pei,[1]
[Affiliations]
[1]School of Medicine, South China University of Technology; Guangdong Provincial People's Hospital/Guangdong Academy of Medical Sciences/Guangdong Provincial Geriatrics Institute, Guangzhou 510080, China
[2]Guangdong Provincial People's Hospital/Guangdong Academy of Medical Sciences/Guangdong Provincial Geriatrics Institute; School of Medicine, South China University of Technology; The Second School of Clinical Medicine, Southern Medical University, Guangzhou 510080, China

[Corresponding author information]
Qiong Ou
[Affiliations]
Guangdong Provincial People's Hospital/Guangdong Academy of Medical Sciences/Guangdong Provincial Geriatrics Institute; School of Medicine, South China University of Technology; The Second School of Clinical Medicine, Southern Medical University, No. 106 Zhongshan Er Rd., Guangzhou 510080, China.
Email: ouqiong2776@hotmail.com

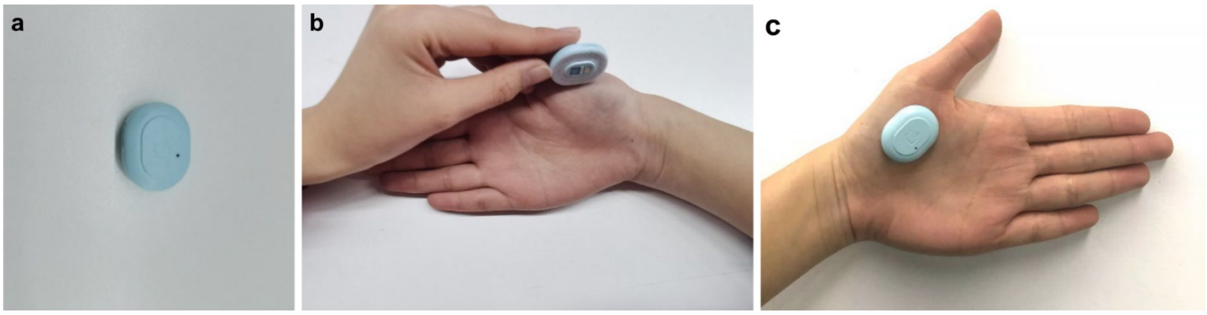

Supplementary Fig. S1 (a) Wearable intelligent sleep monitor (b and c) show the wear location of the device on the palmar thenar major muscles

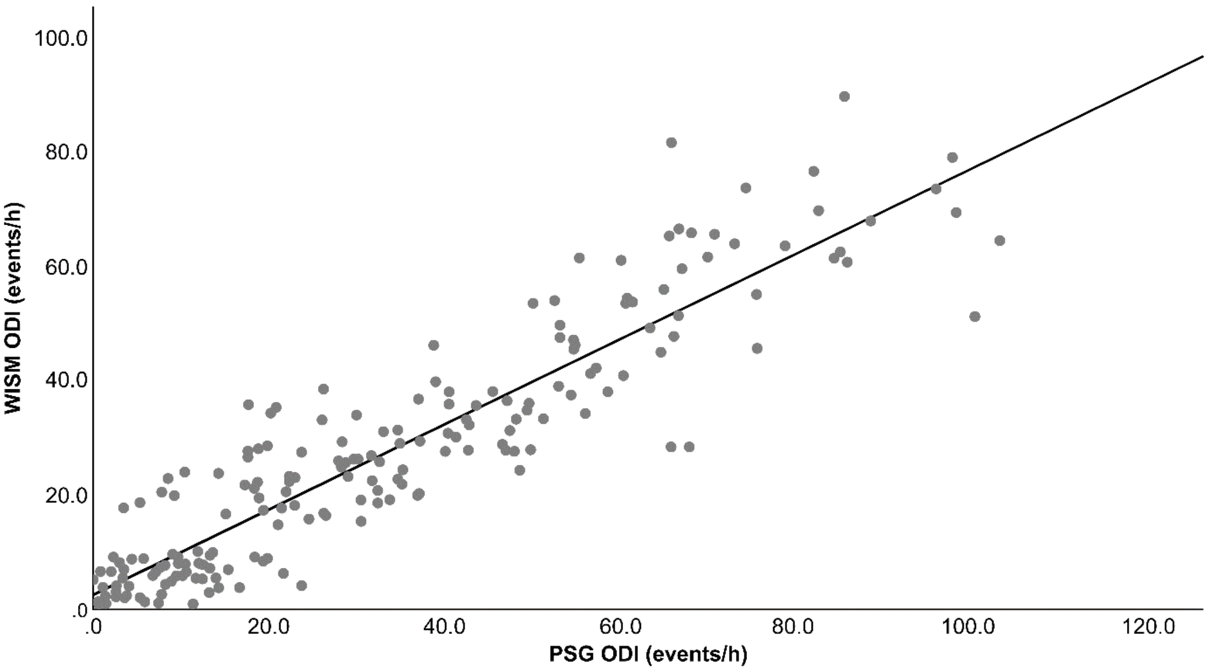

Supplementary Fig. S2 Linear correlation analysis of the WISM and PSG ODIs ($R^2 = 0.845$, $P < 0.001$)

ODI, oxygen desaturation index; WISM, wearable intelligent sleep monitor; PSG, polysomnography

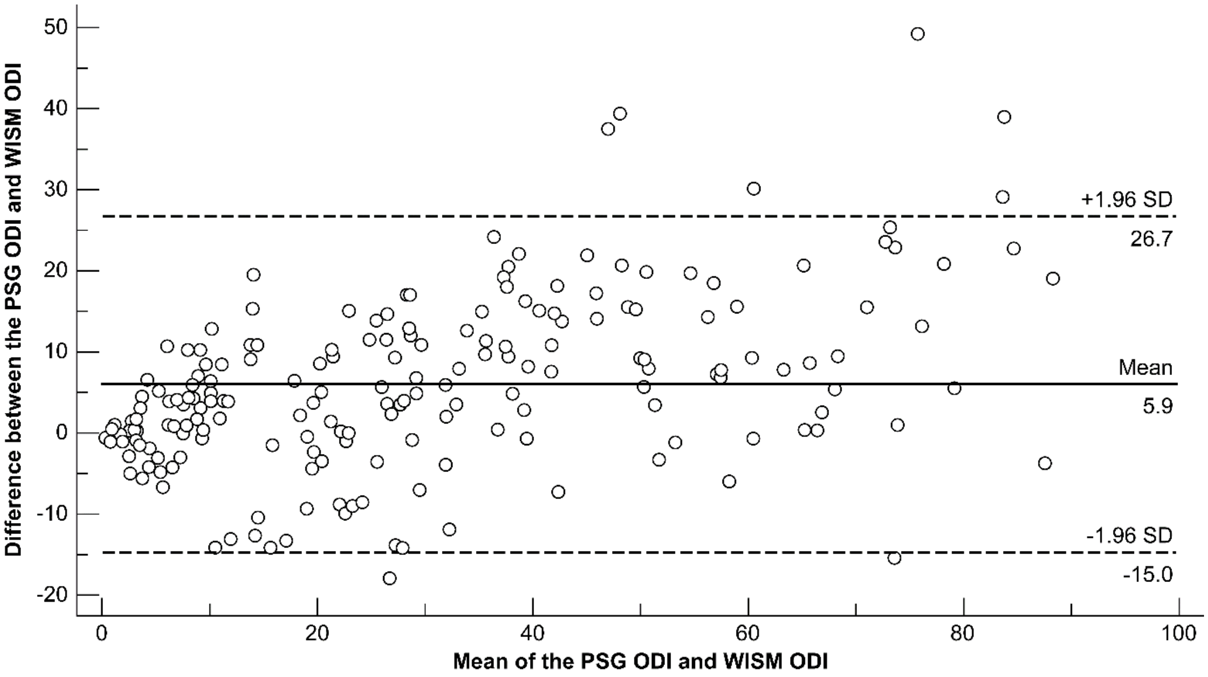

Supplementary Fig. S3 Bland–Altman consistency test results for the ODI calculated from the WISM and the ODI calculated from the PSG. The mean difference between the ODI from the WISM and ODI from the PSG was 5.9 (consistency limit: -15.0–26.7 [N = 196)]

ODI, oxygen desaturation index; AHI, Apnea–Hypopnea Index; PSG, polysomnography; WISM, wearable intelligent sleep monitor
